# Efficient scheduling of multiple software projects for work continuity and identical completion time

**DOI:** 10.1016/j.mex.2025.103215

**Published:** 2025-02-21

**Authors:** Abdulrahman Aldhubaiban, Ali AlMatouq

**Affiliations:** Department of Engineering Management, Prince Sultan University, Riyadh, PO box 66863 Rafha Street, Riyadh, 11586, Riyadh, Saudi Arabia

**Keywords:** Software project scheduling, Multi-objective optimization, Mathematical model, Automatic software management, Efficient Scheduling of Multiple Software Projects for Work Continuity and Identical Completion Time

## Abstract

In software development projects, it is desired to complete multiple projects at minimum cost and time while ensuring that the completion date is the same for all projects to meet certain operational and strategic objectives. Also, full-time employees assigned to projects should be reallocated smoothly to other tasks without any idle time during project execution to minimize costs even further. This study describes a model that enables the use of efficient continuous variable nonlinear solvers for finding the optimal schedule for possibly a large number of multiple software projects that make use of shared resources. The study validates the proposed solution using a random generator of multiple software project instances while interfacing to online optimization solvers to find a solution. Our continuous variable model was solved in the cloud for optimality for large instances of upto 40 different software projects and 100 employees in less than 21 min using nonlinear programming algorithms.

• A continuous variable nonlinear model is developed to efficiently schedule large-scale software projects.
• The model enables scheduling for multiple projects with identical completion times while ensuring work continuity.
• A cloud-based program architecture is designed to facilitate the testing of multiple solvers online.

## Graphical abstract

**Figure:**
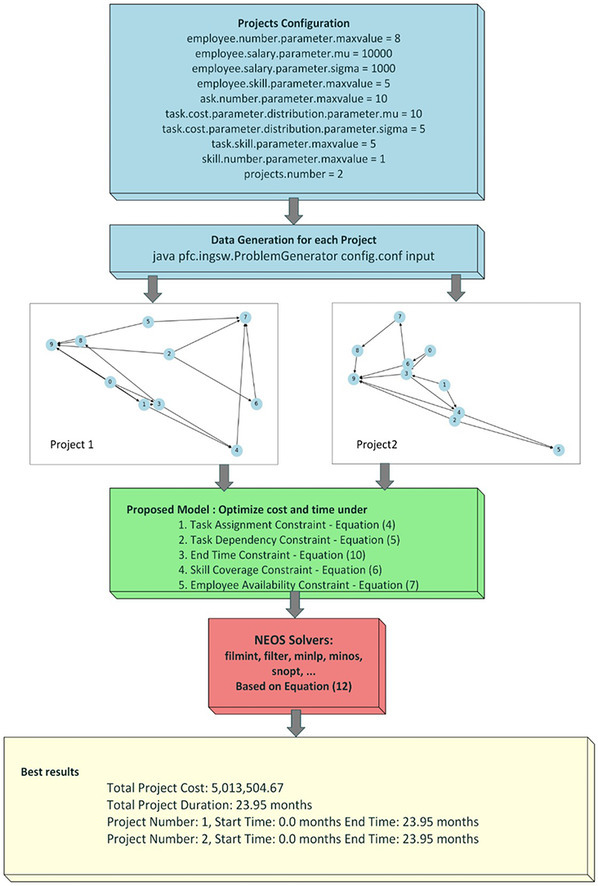

Specifications table

**Table:** 

Subject area:	Operations Research
More specific subject area:	Multi-Project Software Project Scheduling
Name of the method:	Efficient Scheduling of Multiple Software Projects for Work Continuity and Identical Completion Time
Name and reference of original method:	Alba, Enrique, and J. Francisco Chicano. ”Software project management with GAs.” Information sciences 177.11 (2007): 2380-2401.[1]

## Introduction

Project management is the backbone of any successful project in an organization. It has four core components: planning, execution, monitoring, controlling, and closing. Project scheduling is part of the planning phase in software projects and optimizing the assignment of employees in software projects can make a significant difference in managing software projects effectively [2]. Scheduling in software projects consists of planning, allocation of shared resources to tasks and timeline development to ensure software projects meet their objectives within the specified constraints. Underestimating or overestimating costs could lead to defects in project delivery, cost overruns and project delays. Creating an employee assignment schedule in software projects is one of the important activities in software development organizations [3] and is known as the Software Project Scheduling Problem (SPSP). It involves finding the best allocation of human resources that will minimize project cost and duration while satisfying project skill requirements.

The surveys conducted in [3], [4] and [5] summarize SPSP research completed in the last decade and reveal that previous attempts focus on different aspects related to the managing of single software project disturbances that could occur and on the algorithmic challenges associated with solving the mathematical models developed. The studies in [6], [7], [8] and [9], for example, address disturbances like unavailability of employees, employee resignations, employee learning curve and uncertain activity duration. The multiple software project scheduling problem studied in [6], [10], [11] and [12] focus on the algorithmic aspects for solving SPSP for multiple projects and on the different project disturbances, but do not address the global requirements needed often for managing multiple projects like identical completion time and minimum employee idle time.

The widely used mathematical representation in [1] captures the important aspects of the SPSP but employs both continuous and binary decision variables which introduces solution complexity. The problem becomes intractable to solve using exact methods, especially for large scale multiple project instances. Hence, Genetic Algorithms (GA) was employed to solve different individual project instances for optimizing project cost and duration. However, GAs are known for not scaling well with complexity and can not provide any theoretical guarantees on reaching the optimum solution (as with any other heuristic technique). Moreover, the original model did not address the important work continuity constraint nor the practical need for terminating multiple projects at the same time.

Software development organizations are often faced with idle time and project gaps when project teams are assigned to multiple projects having different completion times. Such idle times and gaps could inflate the cost of projects and may not permit the smooth reallocation of shared resources. For example, during idle times, there could be leasing of unutilized cloud infrastructure when some projects are completed and some still active and increase in operational costs when full-time employees are waiting for a task to be completed. Completing multiple software projects at the same time helps to reduce these costs and meet additional operational objectives like consolidating financial budgeting of resources; avoiding the repetition of post-project activities and satisfying, for example, some marketing strategies that demand releasing multiple products at the same time.

In summary, the majority of the models developed for solving the SPSP problem required heuristic/evolutionary algorithms, as in [1] and [11], since the problem becomes intractable when large sets of binary variables are employed. There was no attempt to formulate a continuous variable nonlinear model representation that can be solved efficiently. Second, the previous studies did not consider the possible operational need for having multiple projects terminating at the same time nor the need for work continuity and assumed no cost associated with employee idle time. The work continuity constraint has been previously studied in the context of general project management problems as in [13] but required the definition of boolean variables, which introduces complexity.

In this study, as compared to previous studies, we design a model that will only employ continuous variables (no integer or binary variable definitions required), which permits the use of efficient nonlinear programming algorithms to the solve the SPSP problem for optimality, even for large scale problems. Furthermore, the model results in generating employee assignment schedules for multiple software projects terminating at the same time while satisfying both minimum cost and minimum project duration criteria and reducing employee idle time. The model is solved in the cloud using a special Python interface that can connect to free internet-based optimization solvers hosted by Network Enable Optimization System (NEOS) platform [14] for the purpose of comparing the performance of different solvers for solving randomly generated problem instances. Our model was solved for optimality for large instances of upto 40 different software projects and 100 employees in less than 21 min. The method is recommended for large-scale software development projects that require synchronized completion, efficient resource utilization, and a focus on minimizing costs and idle time for large scale multiple projects.

The first section discusses the research design and methods, emphasizing the model proposed; the use of different state of the art solvers available from NEOS [14] and the method of randomly generating the software project instances. The methods are validated in the following section that explains the experimental configuration. Finally, the study concludes by highlighting the key contribution of the work, its limitations, and implications for further work.

## Method details

The first section will review the optimization model discussed in [1] which formulates the baseline SPSP that minimizes project cost and duration. We do a simple reformulation and add a new objective which will allow finding a schedule that will minimize also employee idle time while ensuring identical completion time of all projects. We then briefly discuss the random project generator discussed in [15] and the architecture of the program used which is interfaced to the online NEOS servers [14].

#### Baseline model with common project completion time

The model consist of three sets representing Employees E; Tasks T and Skills S. Additionally, it incorporates four variables: $t_{start,t}$, representing the starting time of task t; $t_{end,t}$ representing the ending time of task t; $a_{et}\in \left[0,1\right]$ representing employee e availability for task t with $a_{et}=1$ reflecting full availability and $x_{et}\in \left[0,1\right]$ representing employee e level of dedication to task t, with $x_{et}=1$ reflecting full employee dedication to task t, where e∈E and t∈T, respectively. Four parameters also influence the model: the salary of employee e, denoted as $e_{e}^{\text{salary}}\in I_{\geq 0}$, a binary parameter that is equal to one when employee e possesses skill s, denoted by $e_{es}^{\text{skills}}\in \left\{0,1\right\}$, a binary parameter that is equal to one when task t requires skill s, denoted by $t_{ts}^{\text{skills}}\in \left\{0,1\right\}$, and a positive number that measures the effort required to complete a task in person-months, denoted by $t_{t}^{\text{effort}}$. These sets, variables and parameters are shown in Table 1. The tasks t∈T are related to all multiple project tasks combined. In addition, a dependency binary matrix of size T×T captures the dependencies between tasks for all projects and is defined as follows [1]:

(1) $$M_{i,j}=\left\{ \begin{array}{cc} 1, & \text{if}\;\;\text{task}\;i\;\text{depends}\;\text{on}\;\text{task}j\\ 0, & \text{Otherwise} \end{array}\right.$$

As in [1], the objective function aims to optimize both the cost and time required to complete the software projects. Specifically, it seeks to minimize the sum of the total time and total cost of employee salaries as follows:

(2) $$\text{Minimize}\;\overset{Project\;Duration}{\overset{︷}{{\text{Max}}_{t\in T}\left(t_{end,t}\right)}}+\gamma \overset{Project\;Cost}{\overset{︷}{\sum_{e\in E}\sum_{t\in T}x_{et}\cdot t_{t}^{\text{effort}}\cdot e_{e}^{\text{salary}}}}$$

where, $t_{end,t}$ is calculated as the start time for a task, denoted by $t_{\text{start},t}$, plus its effort (in person-months) divided by the sum of employee assignments as follows [1]:

(3) $$t_{\text{end},t}=t_{\text{start},t}+\frac{t_{t}^{\text{effort}}}{\sum_{e\in E}x_{et}}$$

The first term in the objective (2) minimizes the total duration in months for all project tasks by minimizing the maximum ending time among all tasks denoted by ${\text{Max}}_{t\in T}t_{end,t}$. The second term minimizes the cost of person months. The tuning parameter γ controls the trade-off between the two objectives.

**Table 1:** Model sets, variables and parameters, e∈E, t∈T and s∈S.

**Set**	**Description**
E	Set of employees
T	Set of tasks
S	Set of skills
**Variable**	**Description**
$x_{et}$	Dedication of employee e to task t in one month (between 0 and 1)
$t_{\text{start},t}$	Starting time of task t in months
$t_{\text{end},t}$	Ending time of task t in months
$a_{et}$	Availability of employee e for task t (between 0 and 1)
**Parameter**	**Description**
$e_{e}^{\text{salary}}$	Monthly salary of employee e
$e_{es}^{\text{skills}}$	Binary indicator of whether employee e has skill s
$t_{ts}^{\text{skills}}$	Binary indicator of whether task t requires skill s
$t_{t}^{\text{effort}}$	Effort needed for task t in person-months

The second term in the objective calculates the cost of all projects by summing the salaries paid to the employees for their dedication to each task in all projects, charged by the amount of months actually worked. Hence, such model will be useful if employees are paid by the hour and will not be appropriate for the cost of full-time employees, especially if the schedule will result in employee idle time waiting for other tasks to complete. Note that the first term in the objective function renders the problem to be a non-convex optimization problem due to the division operation by the decision variable $x_{et}$. Without loss of generality, the study will assume that all projects will start at the same time.

The following are the model constraints as in [1]:

1. **Task Assignment Constraint:** Each task must be assigned to at least one equivalent employee: [1]
  (4) $$\sum_{e\in E}x_{et}\geq \epsilon \;\forall t\in T,\epsilon \ll 1$$
2. **Task Dependency Constraint:** Task $t_{i}$ must start only after the dependencies of task $t_{j}$ are completed:
  (5) $$t_{\text{start},t_{i}}\geq t_{\text{end},t_{j}}\;\forall t_{i},t_{j}\in T\text{,}\;\text{and}\;M_{i,j}=1$$
3. **Skill Coverage Constraint:** The combined skills of assigned employees must meet or exceed the skills required by the task:[1]
  (6) $$\sum_{e\in E}e_{es}^{\text{skills}}\cdot x_{et}\geq t_{ts}^{\text{skills}}\;\forall t\in T,\forall s\in S$$
  This constraint ensures that at least one person-month is available that possesses the necessary skills needed to complete each task. This number can be increased if desired by making $t_{ts}^{\text{skills}}$ more than one.
4. **Employee Availability:** The availability of an employee must reflect their assignment to tasks: [1]
  (7) $$a_{et}\geq 1-\left(t_{\text{end},t}-t_{\text{start},t}\right)\cdot x_{et}\;\forall e\in E,\forall t\in T$$
  This inequality will serve to measure the availability of each employee during each month.
5. **Positivity and Box Constraints:**
  (8) $$0\leq x_{et}\leq 1,\;\;\;0\leq a_{et}\leq 1,\;\;\;t_{start,t},t_{end,t}\geq 0,\forall e\in E,t\in T$$

#### Synchronizing project completion times

To enable obtaining solutions with identical project completion times, all multi-project tasks are considered to be part of one entire project that incorporates all single project dependencies and the min(max(·)) objective in (2) is reformulated by introducing a new variable z and a new constraint as follows [16]:

(9) $$\begin{array}{cc} & \text{Minimize}\;z+\gamma \sum_{e\in E}\sum_{t\in T}x_{et}\cdot t_{t}^{\text{effort}}\cdot e_{e}^{\text{salary}}\\ & \text{subject}\;\text{to}\\ & z\geq t_{end,t},\;\;\;\forall t\in T,\;\text{and}\;\text{subject}\;\text{to}\;\text{constraints}\;\text{(4)-(7)} \end{array}$$

The above formulation will allow finding the minimum synchronized project completion time for completing all projects. In other words, all projects will terminate at the same time when an optimal solution is obtained since z is a common lower bound for all project tasks. Completing some projects earlier than other projects will require assigning more employees to that project which will inflate the cost. This is demonstrated also in the experiment section of this study. If individual completion times are desired, than each project can have a separate lower bound to be minimized.

#### Employee idle time

As discussed earlier, it may also be desired to find a schedule that reduces employee idle time as much as possible so that the costs reflects full-time employee costs. This will be done simply by minimizing the difference between the duration of each project and the total time the employee worked during the entire duration of that project (in months) defined as employee idle time:

(10) $$idle_{e}=\left({\text{Max}}_{t\in T}t_{end,t}-\sum_{t\in T}x_{e,t}t_{dur,t}\right),\forall e\in E$$

where, $idle_{e}$ denotes employee e idle time and $t_{dur,t}$ is duration of the project and can be found using the below equation:

$$t_{dur,t}=\frac{t_{t}^{\text{effort}}}{\sum_{e\in E}x_{et}}$$

Hence, the new objective will become:

(11) $$\text{Minimize}\;\overset{Project\;Duration}{\overset{︷}{{\text{Max}}_{t\in T}\left(t_{end,t}\right)}}+\overset{Project\;Cost}{\overset{︷}{\gamma \sum_{e\in E}\sum_{t\in T}x_{et}\cdot t_{t}^{\text{effort}}\cdot e_{e}^{\text{salary}}}}+\overset{All\;Employee\;idle\;time}{\overset{︷}{\sum_{e\in E}idle_{e}}}$$

Introducing the variable z as before, we obtain the following objective:

(12) $$\begin{array}{cc} & \underset{z,x_{et},a_{et},t_{start,t},t_{end,t}}{\text{Minimize}}\;z+\gamma \sum_{e\in E}\sum_{t\in T}x_{et}\cdot t_{t}^{\text{effort}}\cdot e_{e}^{\text{salary}}+\sum_{e\in E}\left(z-\sum_{t\in T}x_{e,t}t_{dur,t}\right)\\ & \text{subject}\;\text{to}\\ & z\geq t_{end,t},\;\;\;\forall t\in T,\;\text{and}\;\text{subject}\;\text{to}\;\text{constraints}\;\text{(4)-(8)}\;\text{as}\;\text{before} \end{array}$$

Consequently, the additional objective will result in finding a schedule that will reduce the idle time for all employees working on all tasks, while ensuring that all projects terminate at the same time. However, this requires fine tuning the trade-off tuning parameter γ to reach this goal as will be demonstrated later in the experiment section.

#### Maximum dedication constraint

In [1], an additional constraint was included to avoid employee overtime as follows:

(13) $$\sum_{t\in C}x_{e,t}\leq e_{e}^{\max},\forall e\in E$$

where, C is the set of concurrent tasks in the final schedule (i.e. tasks overlapping in time), and $e_{e}^{\max}$ is the maximum dedication per employee which is usually equal to 1 if no overtime is permitted. We note that constraint 13 renders the SPSP a combinatorial problem that is difficult to solve since the set C requires the definition of a large number of switching binary variables and associated constraints so that no employee works more than what he is capable of. This study will not include constraint 13 in the final model, instead the following simplifying assumption will be used.

Assumption 1 If constraint 13 is not satisfied for any employee e∈E for a certain set of concurrent tasks t∈C, then additional employees with the same required task skills and approximate salaries are available to cover the extra load.

This is not a limiting assumption in practice if there are employees with the same set of skills and salaries. These employees can be counted as one employee for the purpose of preparing project schedules. The solution of the optimization problem can then determine if additional employees of the same set of skills and approximate salaries are required or not.

Another technique to satisfy constraint (13) would be to resolve (12) with a different value of the tuning parameter μ. Increasing the value of γ will emphasize cost and de-emphasize project duration and idle time objective in the model, which permits increased project duration in the optimal solution and the chances for the satisfaction of (13). In other words, the longer the completion time of all projects, the more time it takes to complete individual project tasks and the less chances will any employee work more than what he is capable of. Using cross validation techniques, at each iteration a certain value of γ can be selected and problem (12) can be resolved until (13) is satisfied. A simple technique for finding the most suitable value for γ can be used by first specifying the maximum number of resolving iterations $i_{\max}$ and then finding a sequence of values $\gamma_{1}=0<\gamma_{2}<\cdots ,<\gamma_{i_{\max}}=\gamma_{\max}$ using a logarithmic scale as follows:

(14) $$\begin{array}{c} \gamma_{i}=\log_{10}10^{\left(i\gamma_{\max}\right)/i_{\max}},\;\;i=2,3,\cdots ,i_{\max}-1 \end{array}$$

Consequently, at each iteration i, the minimization problem (12) is resolved using $\gamma_{i}$ until (13) is satisfied.

In fact, the second objective in (12) promotes solution sparsity since the positive weighted sum of the decision variables $x_{et}$ is the positive weighted $\ell_{1}$ norm; i.e. $\sum_{e\in E}\sum_{t\in T}x_{et}e_{e}^{sallary}={∥e_{e}^{sallary}\cdot x_{et}∥}_{\ell_{1}},\;\;\;\;\;x_{et}\geq 0$. This penalty is often used in compressive sensing techniques to find a solution vector with a small number of nonzero values in $x_{et}$ [17].

#### Program architecture

We develop an optimization model using Python linked with a Java package named ”pfc.ingsw.ProblemGenerator” developed in [15], for generating random instances of software projects. A configuration file also simulates employees’ skills and salary information. This is then used to create random instances of software project tasks with random task dependencies, random skill requirements and random employee skill possession.

NEOS (Network Enabled Optimization Solvers) is a suite of powerful optimization tool designed to solve linear and non-linear optimization problems in the cloud [14]. Using NEOS set of different solvers interfaced with Python, a solution is obtained to verify that a consistent answer is obtained. The results are then displayed on project time-line plots and employee dedication and availability charts. The solvers used in this study are described below: [14]

• **MINOS**: Modular In-core Nonlinear Optimization System - Solver for large-scale linear and nonlinear programming using a reduced gradient algorithm. [18]
• **SNOPT**: Sparse Nonlinear Optimizer - A solver for large-scale nonlinear programming problems, using sequential quadratic programming (SQP). [19]
• **FILMINT**: Based on the LP/NLP algorithm by Quesada and Grossmann implemented in a branch-and-cut framework [20].

**Fig. 1:**
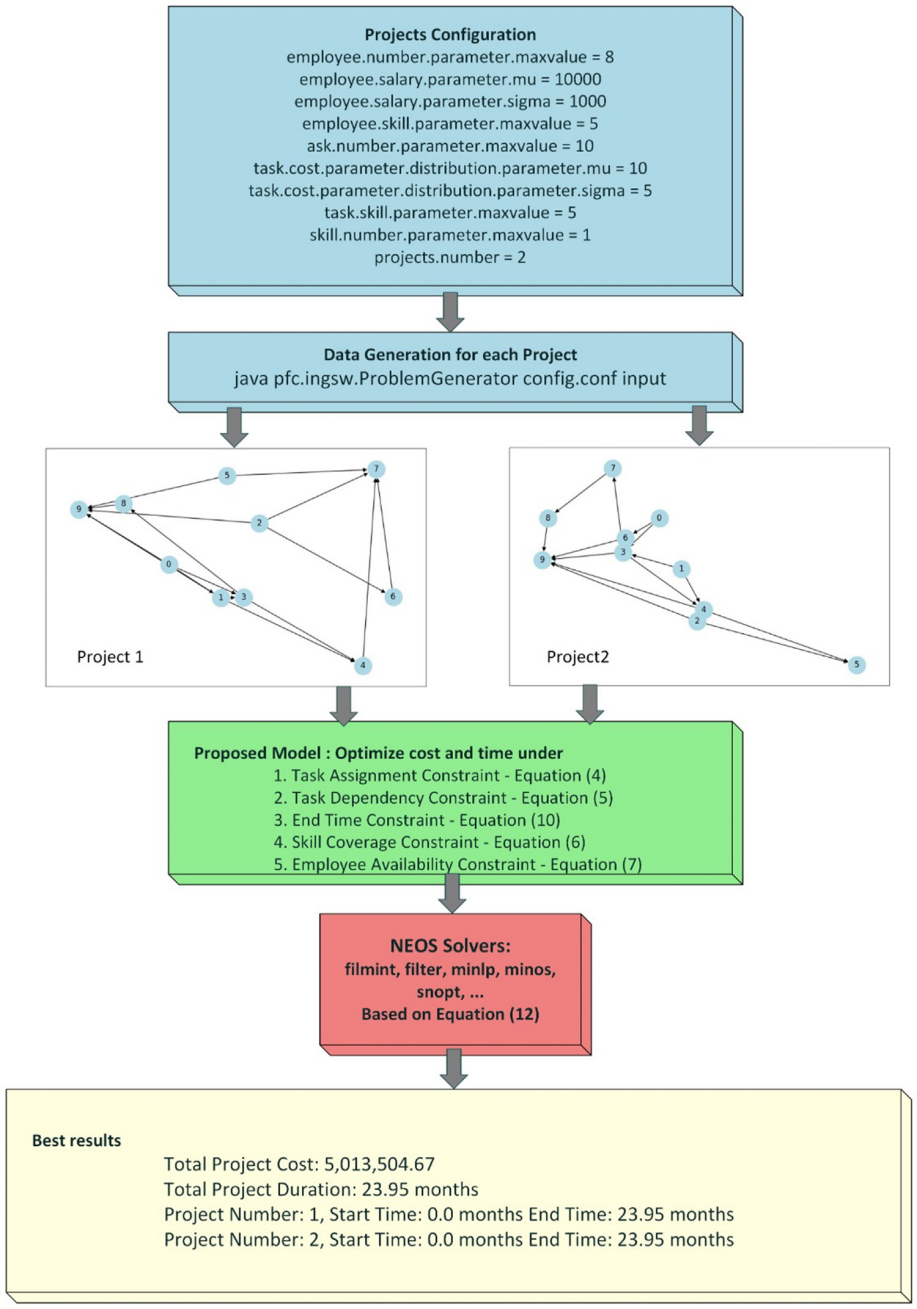
Program Architecture.

Although, FILMINT was designed for solving mixed integer nonlinear programs, we found that this algorithm in fact solves large instances of the SPSP problems very well. Fig. 1 illustrates the detailed architecture of the program, which includes project configuration, random data generation, model optimization and result generation. The project configuration phase sets the basic parameters for each project pertaining to the number of employees, their salary distributions, their skill levels, and the characteristics of the tasks for each project and their corresponding dependencies. The number of full-time employees can be set with their associated salaries. The random generator will generate salaries according to a predetermined normal distribution. Tasks are defined according to their quantity, cost, and skill sets, all generated randomly assuming a uniform distribution with a specified range.

Following the configuration phase, we organize the input data for each project utilizing the previously established parameters. We achieve this by executing the specified command-line instruction: java pfc.ingsw. The pfc.ingsw.ProblemGenerator class will presumably trigger a problem generator to build the necessary data used at later stages of optimization. Such data forms the basis upon which one can model and solve an optimization problem. The data set includes task assignment, task dependency, end-time, skill coverage, and employee availability.

## Method validation

### Experiment #1

This section presents the results of applying our approach to scheduling two distinct projects to simplify analysis and presentation. The input configuration for both projects are as follows: Number of Employees: 8, Salary (mean, 10,000 with standard deviation of σ=1000. Employee Skill Level: 5. Task Parameters: Number of Tasks per project: 10. Task Cost Distribution (mean, 10, standard deviation σ=5, Maximum Skill Requirement: 5.

Two projects exhibiting task dependencies are randomly generated as shown in Fig. 1. These dependencies, along with the model constraints detailed in subsection 3, form the basis for testing different optimization techniques. The objective is to find the best possible total cost and duration for each project while ensuring identical completion time and minimum employee idle time.

Table 2 summarizes the results obtained from applying these optimization algorithms for solving the model presented in Section III for the two considered projects. The maximum dedication for each employee was set to more than 5. Both FILMINT and MINOS achieved identical outcomes and the solution was obtained in less than 30 s demonstrating the efficiency of the model. The total project cost of 5,013,504.67 and a synchronized completion time of 23.95 months for both projects. SNOPT also found a feasible solution, albeit with a slightly higher cost (5,013,510.98) and a marginally longer duration, possibly due to differences in solver precision.

**Table 2:** Results of the used optimization techniques.

Optimization Technique	Total Cost	Total Duration	Project 1 Total Duration	Project 2 Total Duration
FILMINT	5,013,504.67	23.95523313 months	23.95523313 months	23.95523313 months
MINOS	5,013,504.6	23.95523312 months	23.95523312 months	23.95523312 months
SNOPT	5,013,510.98	23.95523873 months	23.95523873 months	23.95523873 months

**Fig. 2:**
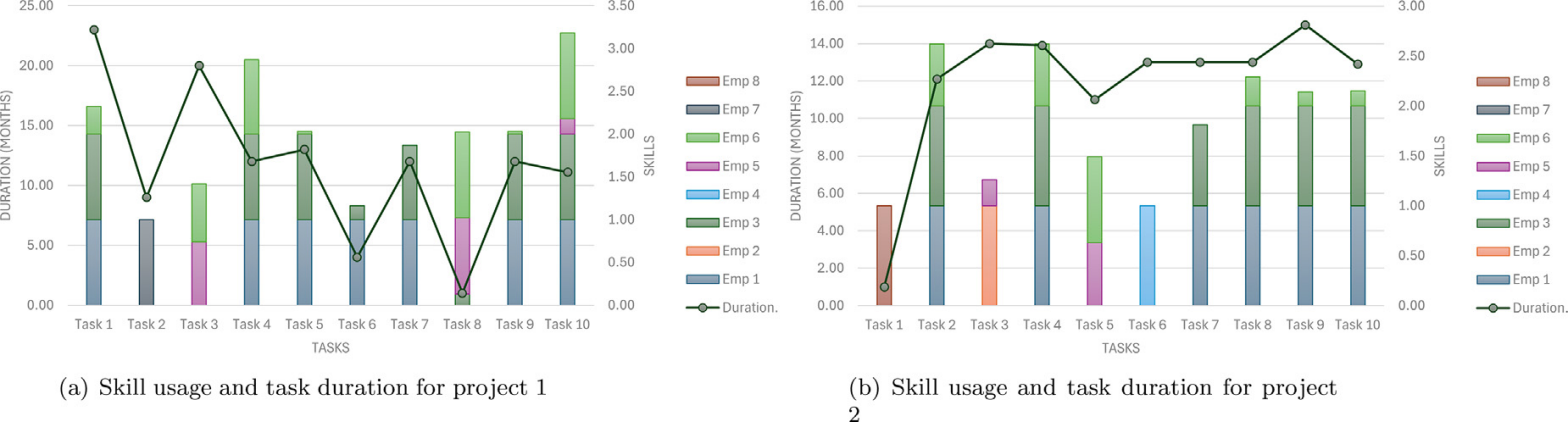
Skill usage and task duration for project 1 (a) and project 2 (b) based on FILMINT.

Fig. 2 shows the duration and skill usage by task for Project 1 and Project 2 using FILMINT optimizer. In Project 1, the bars represent the duration each employee spent on different tasks, while the dashed lines show the skill levels (normalized between 0 and 1) required for each task. Higher skill levels correlate with certain tasks where employees have invested more time, such as Task 1 and Task 5, indicating that these tasks may have required specialized expertise. Employee 3 exhibits consistently high skill levels across tasks, reflecting a significant involvement in skill-demanding tasks. Project 2 shows a similar pattern. Tasks like Task 3 and Task 6, which have higher skill requirements, require more hours from employees. Employee 4’s high skill level on Task 6 corresponds to a higher duration, suggesting a concentrated effort in specialized tasks. Employees 1 and 5 also demonstrate considerable time contributions aligned with skill requirements, emphasizing their roles in this project.

**Fig. 3:**
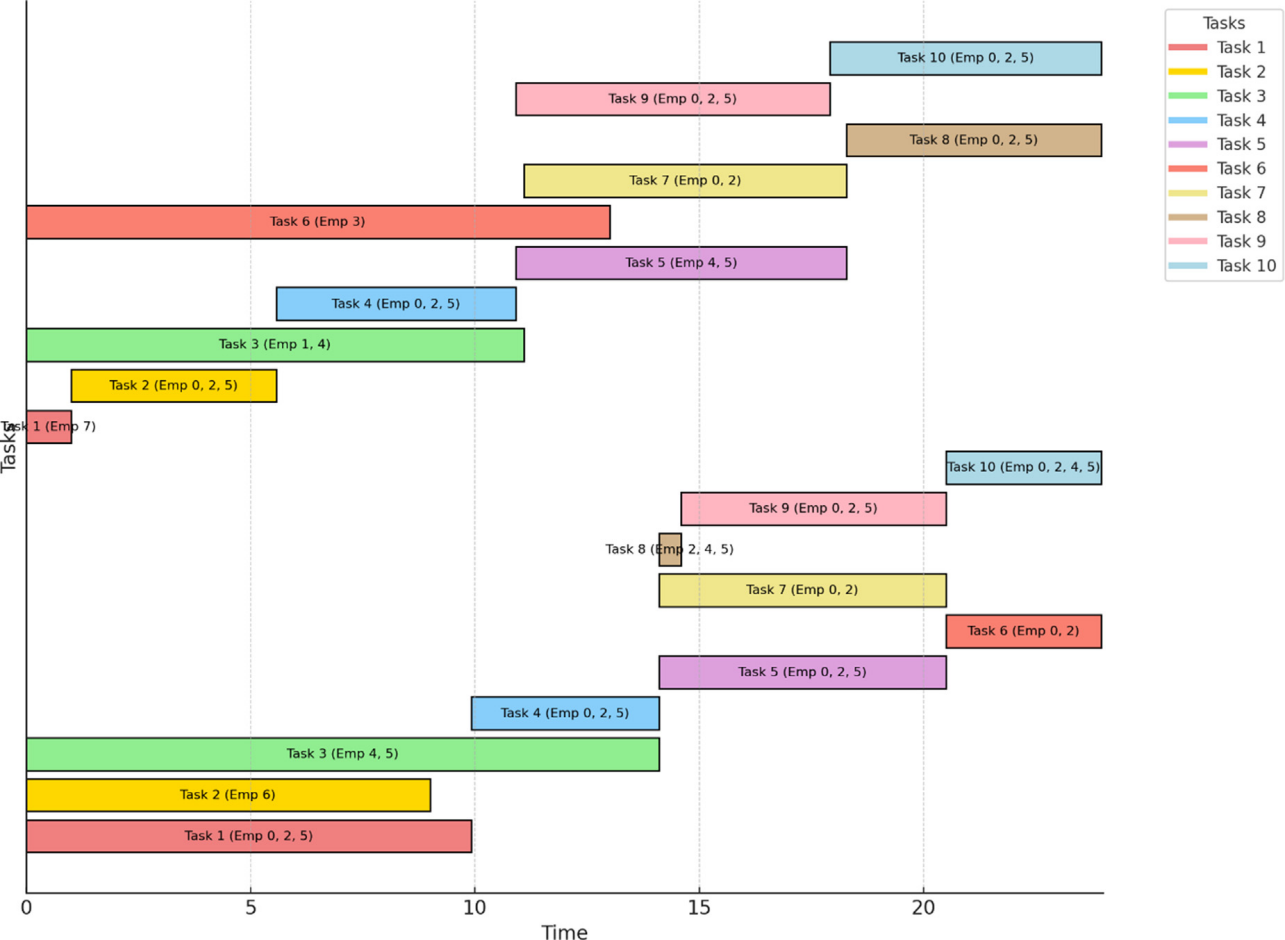
Task Timeline for Project 1 and Project 2 (with Employee Assignments) based on FILMINT optimizer.

The timeline graph is shown in Fig. 3 that aids in showing the cross-project tasks with duration, and the assignment of employees for each task. Each horizontal bar corresponds to a specific task within either Project 1 or Project 2, with start and end points marking the task’s duration. The project timeline clearly shows that both projects will terminate at the same time and employees are allocated to work on both projects.

**Fig. 4:**
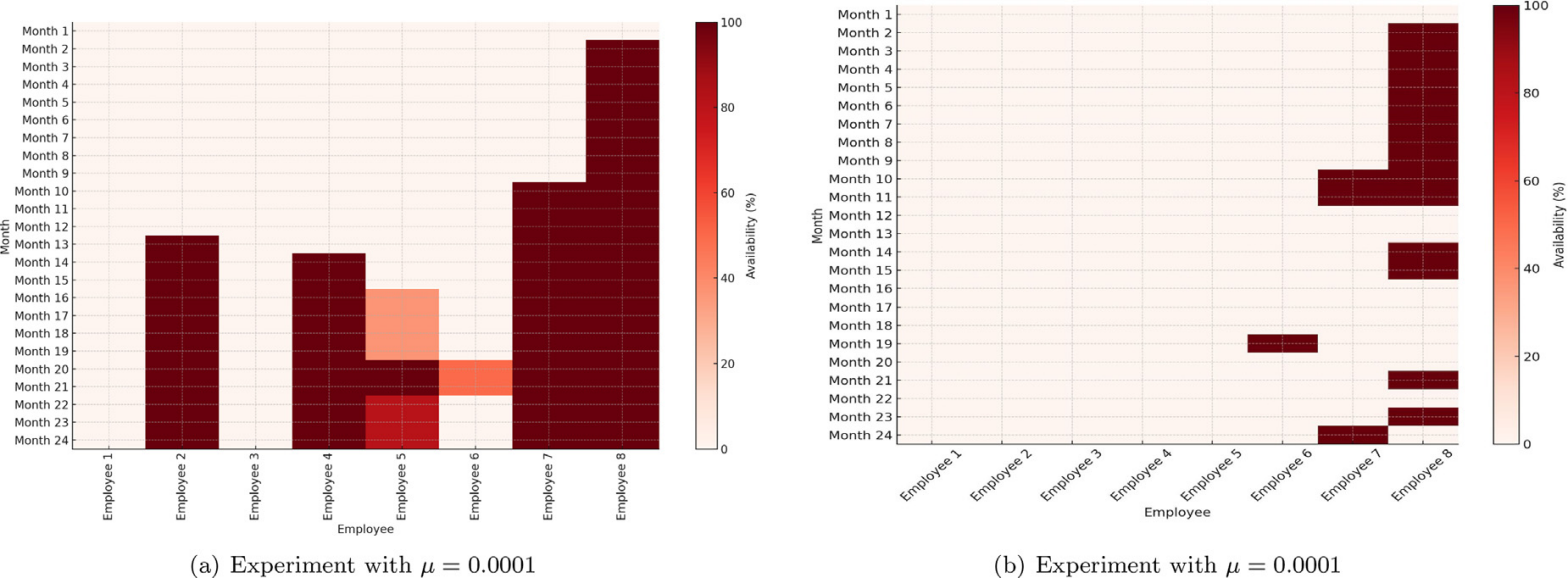
Employee availability heat-map: (a) Experiment with μ=0.001, (b) Experiment with μ=0.0001.

The heat-map results shown in Fig. 4 demonstrate employee availability. It shows that there is high availability and hence large idle time in this result. This was attributed to the tuning parameter used μ=0.001 which resulted in emphasizing on lower cost assuming employees are charged by the number of months actually working. Hence, the calculated costs does not truly reflect the total project cost if full-time employees are employed for these two projects. The actual cost is the cost of the salaries of all employees for the entire duration of the project. Consequently, we repeated the same experiment using the same parameters described earlier but with a reduced value of the tuning parameter μ to be μ=0.0001. The results are summarized in Table 3. The cost of the project increased by 138% but the project duration decreased by 34.4% and the idle time significantly decreased by more than 80%. Hence, this demonstrates that the model was able to find a schedule with both minimum cost and project duration, while ensuring that both projects have the same duration and idle time is minimized. However, this came at the expense of not satisfying constraint (13) for some employees. As mentioned earlier, if additional employees with the same set of skills and approximate salaries are available to cover the extra load in the overlapping tasks, then the obtained solution is still applicable in practice. Otherwise, cross validation can be used by resolving the minimization problem using a grid of values for μ using (14) until a satisfactory solution is obtained.

**Table 3:** Results of repeated experiment with μ=0.0001.

Optimization Technique	Total Cost	Total Duration	Project 1 Total Duration	Project 2 Total Duration
FILMINT	11,910,121	15.698 months	15.698 months	15.698 months

#### Experiment #2

This experiment will demonstrate the efficiency of the proposed model for solving relatively large problem instances. We have used the same parameters described in the previous experiment but varied the minimum number of skills per employee to be 2 and the maximum to 4 and the maximum skill value set to be 4. Consequently, to test efficiency of the model, the number of projects, the number of employees and the number of tasks per project for four randomly generated large multi-project instances were solved as shown in Table 4. The algorithm used in all experiments was FILMINT which provided stable performance compared to MINOS and SNOPT. The tuning parameter used was μ=0.00001 in all experiments to emphasize more on reducing employee idle time. The results in Table 4 show the computation time for each problem instance which reflects the efficiency of the technique. Moreover, all projects had exactly the same completion time. We could not solve instances beyond 40 projects and 100 employees due to restrictions on the NEOS server and is not a limitation of the technique.

**Table 4:** Results of Experiment #2.

Num. of Projects	Num. of Employees	Tasks/Project	Synchronized Time (months)	Comp. Time (mins)
5	50	10	18.52	0.5
10	100	10	16.97	5.2
20	50	5	18.22	3.2
30	70	5	17.66	7.1
40	100	5	17.67	20.5

## Conclusion

This paper demonstrates a simple technique for solving the Software Project Scheduling Problem for a large number of multiple projects, taking into consideration cost, project duration, task dependencies, employee skills and task skill requirements while ensuring identical project completion times for all projects. Furthermore, the solution allows schedulers to make the compromise between employee idle time and project cost through the tuning of a single parameter. For complex scheduling problems characterized by large number of projects and employees, this study develops a model that can be solved using generic nonlinear programming solvers, like sequential quadratic programming and reduced gradient solvers that converge to the optimal solution. Future work can address issues related to the uncertainty of employee skill level and task requirements using, for example, robust non-convex optimization techniques. The research develops a framework in which continuous nonlinear optimization algorithms with guaranteed performance can be used to solve large software project scheduling problems.

## Limitations

As mention earlier, not including the maximum employee dedication constraint in (13) is in general not a limiting assumption in practice unless there are no additional employees with the same set of skills and salaries. However, using cross validation techniques the model in (12) can be resolved with a range of values of the tuning parameter μ efficiently until the maximum employee dedication constraint is satisfied.

## Ethics Statement

The authors of this work have read and complied with the ethical requirements for publication in MethodsX and the current work does not involve human subjects, animal experiments or data collected from social media platforms.

## Declaration of completing interest

The authors declare that they have no known competing financial interests or personal relationships that could have appeared to influence the work reported in this paper.

## CRediT authorship contribution statement

**Abdulrahman Aldhubaiban:** Investigation, Software, Data curation, Formal analysis, Visualization, Writing – original draft. **Ali AlMatouq:** Supervision, Conceptualization, Methodology, Writing – review & editing.

## Data Availability

Software program is made available on GitHub Repository.
